# Impact on the Nutritional Status and Inflammation of Patients with Cancer Hospitalized after the SARS-CoV-2 Lockdown

**DOI:** 10.3390/nu14132754

**Published:** 2022-07-02

**Authors:** Patricia Yárnoz-Esquíroz, Ana Chopitea, Laura Olazarán, Maite Aguas-Ayesa, Camilo Silva, Anna Vilalta-Lacarra, Javier Escalada, Ignacio Gil-Bazo, Gema Frühbeck, Javier Gómez-Ambrosi

**Affiliations:** 1 Department of Endocrinology and Nutrition, Clínica Universidad de Navarra, 31008 Pamplona, Spain; lolazaran@unav.es (L.O.); maiteaguas.nutricion@gmail.com (M.A.-A.); csilvafr@unav.es (C.S.); fescalada@unav.es (J.E.); gfruhbeck@unav.es (G.F.); 2 Navarra Institute for Health Research, IdiSNA, 31008 Pamplona, Spain; igbazo@unav.es (I.G.-B.); jagomez@unav.es (J.G.-A.); 3 Department of Oncology, Clínica Universidad de Navarra, 31008 Pamplona, Spain; chopitea@unav.es (A.C.); avilaltal@unav.es (A.V.-L.); 4 Centro de Investigación Biomédica en Red Fisiopatología de la Obesidad y Nutrición (CIBEROBN), 31008 Pamplona, Spain; 5 Centro de Investigación Biomédica en Red de Cáncer (CIBERONC), 31008 Pamplona, Spain; 6 Program in Solid Tumors, Center for Applied Medical Research, 31008 Pamplona, Spain; 7 Metabolic Research Laboratory, Clínica Universidad de Navarra, 31008 Pamplona, Spain

**Keywords:** SARS-CoV-2 lockdown, nutritional status, oncology patients, mortality, inflammation, obesity paradox

## Abstract

Many studies have demonstrated that malnutrition has a negative impact on quality of life and mortality in patients with cancer. During the SARS-CoV-2 lockdown, dietary intake changes were detected in the Spanish population, reflecting an increase in the consumption of fruit, bread, flours, and eggs. The present study analyzed the nutritional status of 728 patients with cancer admitted once the SARS-CoV-2 lockdown finished, comparing it with the previous year as well as with mortality rates. The Malnutrition Universal Screening Tool (MUST) was applied in the first 24 h after admission. Age, gender, days of stay, circulating concentrations of albumin, cholesterol, *C*-reactive protein (CRP), lymphocytes, prealbumin, and mortality data were analyzed. Patients with cancer admitted between June and December of 2020 exhibited no statistical differences in BMI, age, or gender as compared to patients admitted in 2019. Statistically significant differences in nutritional status (*p* < 0.05), albumin (*p* < 0.001), and CRP (*p* = 0.005) levels regarding lockdown were observed in relation with a small non-significant reduction in mortality. In conclusion, following the SARS-CoV-2 lockdown, an improved nutritional status in cancer patients at admission was observed with a decrease in the percentage of weight loss and CRP levels together with an increase in albumin levels compared to oncological patients admitted the previous year.

## 1. Introduction

The prevalence of malnutrition in patients with cancer ranges between 30–70% depending on the malnutrition-screening tool selected, the type of cancer, the clinical situation, and the treatment administrated [1,2]. An awareness of the importance of nutritional status in hospital settings began more than 40 years ago [3]. In this line, the PREDyCES (Prevalence of Hospital Malnutrition) study analyzed the economic impact of hospital malnutrition and the cost of longer hospital stays according to the prevalence of hospital malnutrition in Spain [4]. In a sub-analysis of this study, the prevalence of hospital malnutrition in patients with cancer was measured for the first time in Spain. It was shown that more than 30% of oncology patients and more than 40% of oncology patients who were ≥70 years old were at risk of malnutrition at admission [5].

In the last nutrition guidelines, the European Society for Clinical Nutrition and Metabolism (ESPEN) expert group has emphasized three key steps to update nutritional care for people with cancer, namely to (i) screen all patients with cancer for nutritional risk early in the course of their care regardless of body mass index (BMI) and weight history; (ii) expand nutrition-related assessment practices to include measures of anorexia, body composition, inflammatory biomarkers, resting energy expenditure, and physical function; and (iii) use multimodal nutritional interventions with individualized plans, including care focused on increasing nutritional intake, decreasing inflammation and hypermetabolic stress, as well as increasing physical activity [6]. The Global Leadership Initiative on Malnutrition (GLIM) ranked the top five criteria to identify malnutrition, including three phenotypic criteria (weight loss, low BMI, and reduced muscle mass) and two etiologic criteria (reduced food intake or assimilation and inflammation or disease burden). To diagnose malnutrition, at least one phenotypic criterion and one etiologic criterion should be present [7].

The 2020 SARS-CoV-2 lockdown led to changes in the dietary habits of the Spanish population [8,9]. The first published studies reported surveys referred by the population about eating patterns and physical activity, with very different results depending on the specific population in which the survey was carried out as well as the country analyzed [10,11,12], highlighting the impact on more vulnerable populations such as patients with eating disorders or obesity [13]. The collapse of the healthcare activity in hospitals made a redistribution of the resources to treat patients affected by SARS-CoV-2 necessary, leaving aside other treatments. The delay in the oncologic treatment due to the pandemic has had an important impact on patients with cancer [14]. Many of the consequences derived from the health crisis attributable to the SARS-CoV-2 pandemic are difficult to measure because long-term effects have not yet fully developed [15,16].

The hypothesis of the study was that the nutritional status of patients admitted to hospital after the lockdown would be deteriorated compared to the nutritional status of patients admitted the previous year because of the SARS-CoV-2 lockdown delays in oncological treatment, decreased physical activity, and worse eating habits during the SARS-CoV-2 lockdown. Therefore, the aim of this study was to evaluate the nutritional status of oncologic patients upon admission in a Spanish tertiary University Hospital after the lockdown in comparison with the data obtained the previous year and to analyze the impact on nutritional biomarkers and mortality.

## 2. Materials and Methods

### 2.1. Patient Selection and Study Design

**Figure 1:**
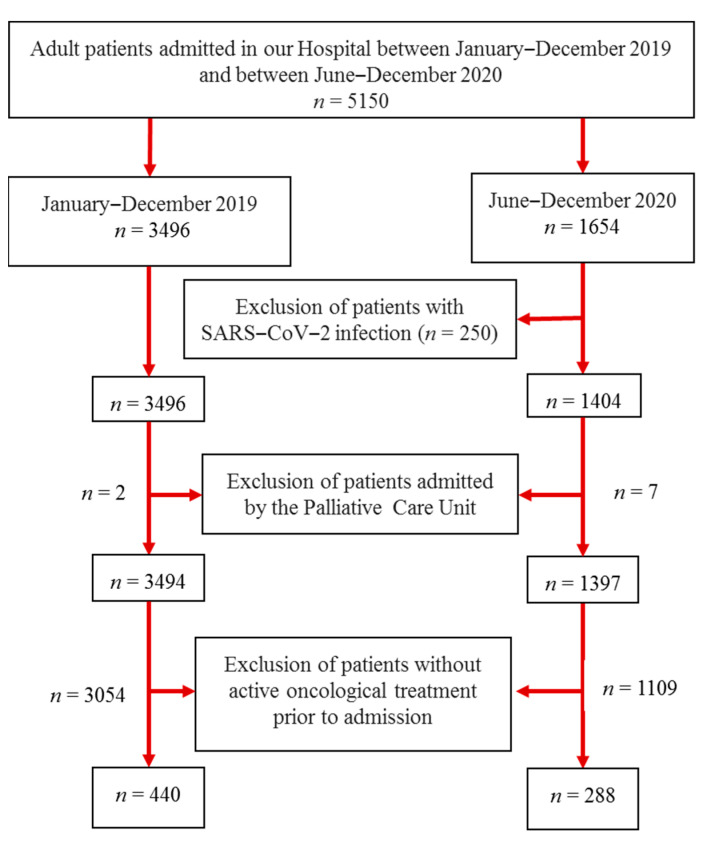
Flow chart describing the patient’s selection criteria.

The study included patients receiving active oncological treatment admitted to the Department of Oncology at the Clínica Universidad de Navarra (Pamplona, Spain), a private tertiary-care hospital with 400 beds, during two different time periods, namely from January to December 2019 as well as from June to December 2020. The patients were admitted due to complications of the treatment or the disease itself; stable patients received treatment without admission. Oncological treatment encompassed different lines of therapy depending on the cancer type, including chemotherapy according to established clinical guidelines as well as novel options in the case of patients enrolled in clinical trials. The SARS-CoV-2 lockdown in Spain started in March and finished in June of 2020. As illustrated in Figure 1, adult patients (age > 18 years) with a diagnosis of cancer prior to the lockdown were included in the study. The study analyzed the nutritional status of patients with cancer admitted after the SARS-CoV-2 lockdown, comparing the nutritional status of patients admitted during both time periods and the impact on mortality. The risk of malnutrition was systematically calculated in the first 24 h after admission using the Malnutrition Universal Screening Tool (MUST) [17]. Patient characteristics such as age, gender, length of hospital stay (LOS), BMI, use of nutritional support, and mortality were analyzed. Mortality data was computed up to 15 months after both periods to avoid mortality bias in the first months after the lockdown.

### 2.2. Lockdown Stage

Following the declaration of a world health emergency by the World Health Organization, the Spanish government adopted a total confinement from 14 March until 21 June 2020. During this SARS-CoV-2 lockdown, essential activities were allowed, which included shopping in supermarkets and grocery stores.

### 2.3. Nutritional Status and Anthropometric Measurements

Following the recommendations of the Consensus Statement of the Academy of Nutrition and Dietetics [18] for intervention of patients with cancer in active treatment, the patient’s responsible nurse registered at admission or within the first 24 h the three variables necessary for the application of MUST, i.e., BMI, percentage of weight loss in the last 6 months, and nutritional alterations due to their clinical situation (Table 1). The MUST was designed to help identify adults who are underweight and at risk of malnutrition. In addition, MUST has been validated as a simple and quick method applicable by any health professional, with a high specificity for early screening, ideally to antedate a comprehensive nutritional assessment [19]. Weight and height of patients was measured by means of the Seca^®^ 704 S column scale (Hamburg, Germany), which automatically calculated the BMI. Furthermore, the patient was asked if he/she had suffered any weight loss in the previous six months, and if the answer was yes, the amount lost (in kg) was recorded, and the percentage of weight loss was calculated. The nutritional alterations derived from their clinical situation and their impact on the nutrient intake for more than 5 days were registered. The alterations were identified in critical patients, those with brain damage, or those who underwent gastrointestinal surgery. According to the MUST protocol, the results allowed to categorize patients in three groups: patients with low, medium, and high risk of malnutrition [17].

### 2.4. Blood Analyses

Blood samples were collected in the morning after an overnight fast and were analyzed in the internal laboratory of the hospital. Total cholesterol concentrations were determined by enzymatic spectrophotometric methods (Roche, Basel, Switzerland). Serum proteins such as albumin and prealbumin have been widely used to determine the patients’ nutritional status [20], and at present, albumin level has been appointed as an etiologic criteria of inflammation in diagnostic tools for malnutrition [7]. Albumin and prealbumin levels were measured using the immunoturbidimetric method (Roche) and immunonephelometry (Siemens, Erlangen, Germany), respectively. Recently, other useful nutritional markers include indicators of inflammation such as *C*-reactive protein (CRP) and total lymphocyte count [21,22]. High-sensitivity CRP was assessed using the Tina-quant^®^ CRP (Latex) ultrasensitive assay (Roche). Lymphocytes were measured using an automated cell counter (Beckman Coulter, Fullerton, CA, USA). Lymphocytes are frequently determined during the hospital stay, but the first value obtained upon admission was taken as the reference.

### 2.5. Statistical Analysis

Sample size was calculated with the G*Power program (version 3.1.9.4, Franz Faul, University of Kiel, Kiel, Germany). Based on previous similar studies with a 0.9 power, a type I error probability associated of 0.05, and expecting a size effect of 0.30 in a two-tailed Student’s *t*-test analysis, at least 235 individuals were needed to be able to reject the null hypothesis. Anticipating a potential loss of participants due to treatment discontinuation or methodological issues, we decided to include at least 250 subjects per group. Data are presented as mean ± SD unless otherwise indicated. CRP concentrations were logarithmically transformed due to their non-normal distribution. The normal distribution of the other variables was adequate for the use of parametric tests. Differences between the two time periods were analyzed by two-tailed unpaired Student’s *t*-tests. Differences in distribution regarding the periods and other variables (gender, MUST, nutritional support, or mortality) were compared by chi-square (χ^2^) analysis. Correlations between two variables were computed by Pearson’s correlation coefficients (r). The calculations were performed using SPSS 23 (SPSS, Chicago, IL, USA) and GraphPad Prism 8 (GraphPad Software, Inc., La Jolla, CA, USA). A *p*-value lower than 0.05 was considered statistically significant. Kaplan–Meier curves were used to analyze the survival data, comparing both groups with Log-Rank test and evaluating the impact of the year of admission, treatment, sex, age, MUST score, and BMI with Cox regression.

## 3. Results

### 3.1. Clinical Characteristics of the Cohort

Anthropometric measurements and biochemical variables are summarized in Table 2. A total of 728 patients with cancer were included in the study: 440 patients were admitted in 2019, while 288 patients were admitted in the 2020 post-lockdown period. The mean age was above 60 years in both time periods, with a higher proportion of men than women (58.2% and 59.0%, respectively). From the whole cohort, 59% were males, and 41% were females, with no differences in gender distribution (*p* = 0.821). The LOS in patients with cancer was higher in those admitted in 2020, almost 1 day more, as compared to patients admitted in 2019 although no statistically significant differences were found. In both groups, the patients were classified according to the tumor stage, with advanced stages (III and IV) accounting for the more frequent cases in both time periods (with a slightly lower number of patients in stages I–III and higher number in stage IV in the 2019 group). The patients were further classified according to monotherapy (chemotherapy) treatment, polytherapy (more than one drug), and other types of treatment (radiotherapy or surgery) (Table 3 and Supplementary Table S1).

### 3.2. Nutritional Status of Patients at Admission

**Figure 2:**
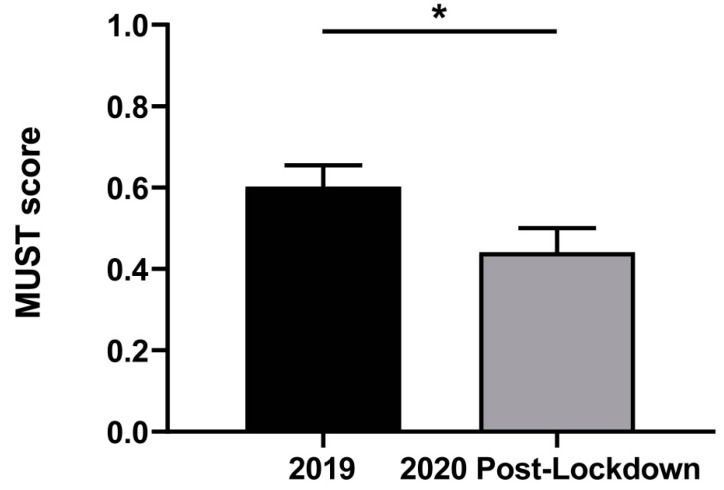
Comparison of nutritional screening score measured with the Malnutrition Universal Screening Tool (MUST) of patients with cancer admitted in 2019 and after the lockdown in 2020. Differences were assessed by two-tailed unpaired Student-s *t*-test; * *p* < 0.05.

**Figure 3:**
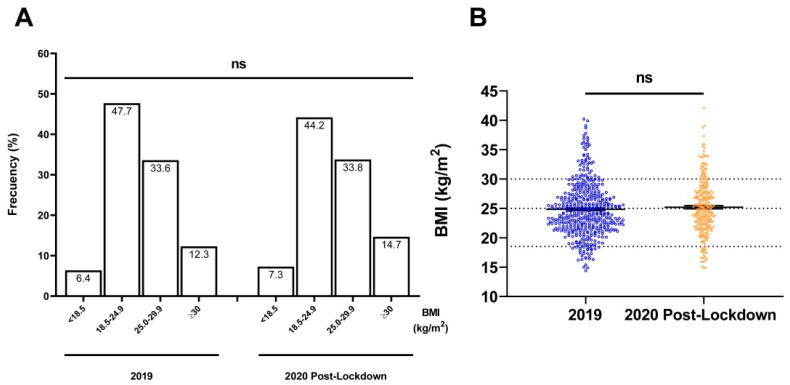
Comparison of body mass index (BMI) (**A**) segregated by weight categories: underweight (<18.5 kg/m^2^), normal weight (18.5–24.9 kg/m^2^), overweight (25.0–29.9 kg/m^2^), and obesity (≥30.0 kg/m^2^) and (**B**) by study periods: patients with cancer admitted in 2019 versus during the post lockdown in 2020. Differences were assessed by χ^2^ (**A**) and two-tailed unpaired Student’s *t*-test (**B**); ns, non-significant.

Lower MUST screening score of patients admitted after the SARS-CoV-2 lockdown was observed as compared to that found in patients admitted in 2019 (Figure 2), with statistically significant differences observed (*p* = 0.042), mostly due to the percentage of weight loss in the last 6 months. The BMI and the impact of the disease on food intake did not show significant differences between the two study periods (Table 2 and Figure 3). No different distribution in the nutritional status of patients according to type of treatment was detected (MUST ≥ 1, *p* = 0.085; MUST ≥ 2, *p* = 0.629), but a higher prevalence of patients with MUST ≥ 2 was observed in the antimetabolites and monoclonal antibodies drugs treatment (40.8% and 19.2%, respectively; *p* = 0.002) and in patients with advanced stages (Supplementary Table S2).

The prevalence of medium risk of malnutrition (MUST = 1) observed in 2019 was higher than that observed in 2020 after the lockdown, 28.9% vs. 20.1% (*p* = 0.008), respectively. The prevalence of high risk of malnutrition (MUST ≥ 2) observed was 20.9% in 2019 and 16.0% in patients admitted in 2020 after the lockdown (*p* = 0.097). No statistically significant differences (*p* = 0.665) in the percentage of patients requiring nutrition support (use of enteral or parenteral nutrition) during the hospitalization in 2019 (9.8%) and 2020 (10.8%) were observed.

Those patients with overweight or obesity are at greater risk for many diseases, including at least 13 types of cancer, than are patients with healthy weight [23]. Cancer related to overweight and obesity between patients admitted in 2019 and 2020 showed different distribution with lower prevalence of patients with obesity-related cancer admitted in 2020 (59.2% vs. 46.4%, respectively; *p* = 0.001). A total of 259 patients in both groups presented cancer related to overweight or obesity, but no statistical significance with mortality was observed (*p* = 0.560); 66.0% of patients with these 13 types of cancer showed MUST score ≥ 2 (*p* = 0.002), in line with the fact that these 13 types of cancer encompass cancers linked with the highest weight loss, such as pancreas, stomach, or colon cancers.

### 3.3. Biochemical Parameters and Inflammation

**Figure 4:**
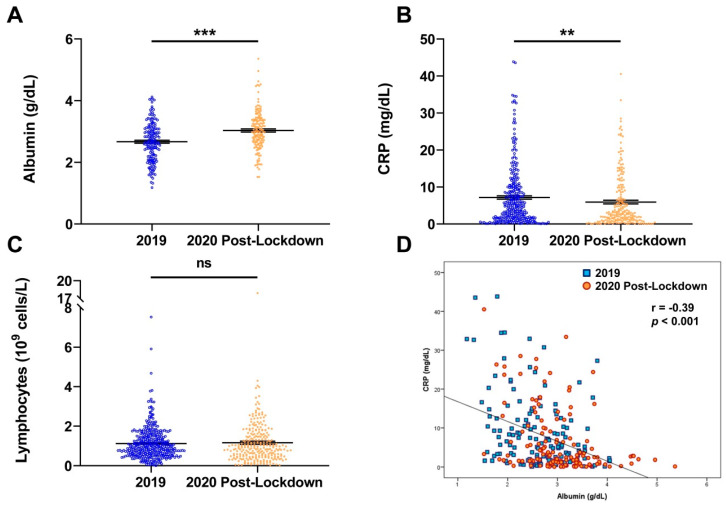
Distribution of circulating albumin (**A**), *C*-reactive protein (CRP) (**B**), and lymphocytes (**C**) as well as the correlation between albumin and CRP levels (**D**) in patients admitted in 2019 and 2020. Differences were computed by two-tailed Student’s *t*-tests (**A**–**C**). Correlation (**D**) analysis was performed by Pearson’s correlation coefficient; ns, non-significant; ** *p* < 0.01; *** *p* < 0.001.

Lymphocytes and CRP levels were routinely measured at admission. Circulating concentrations of CRP measured at the moment of admission in oncological patients in 2019 were significantly higher (*p* = 0.005) than the levels of patients admitted in 2020. On the other hand, albumin concentrations were significantly higher (*p* < 0.001) in patients admitted in 2020, whereas no significant differences (*p* = 0.621) in lymphocyte levels were observed (Figure 4A–C).

Patients with cancer reportedly exhibit a stereotypical acute-phase protein response with CRP increasing and albumin falling, which is maintained across different tumor types [24]. In this sense, a significant (*p* < 0.001) negative correlation between CRP and albumin levels in patients admitted in 2019 and 2020 after the lockdown was observed (Figure 4D). When segregated by time period, the correlations between CRP and albumin levels were maintained (*r* = −0.35; *p* < 0.001 and *r* = −0.36; *p* < 0.001) for 2019 and 2020 after the lockdown, respectively.

### 3.4. Mortality

**Figure 5:**
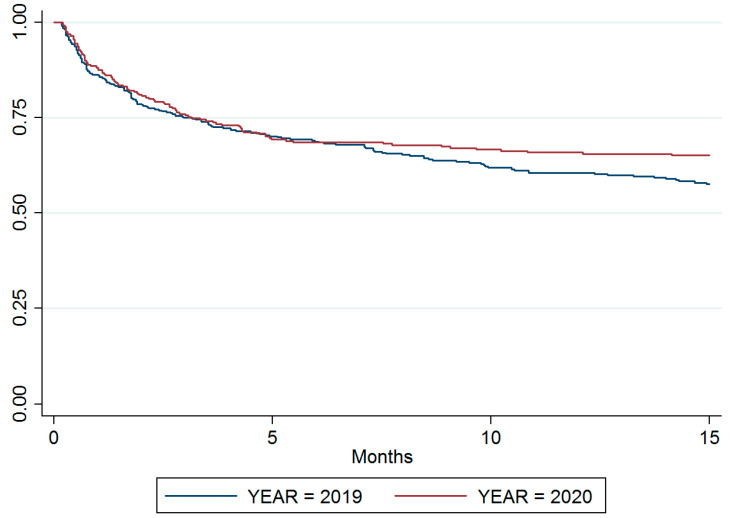
Kaplan–Meier plot. Survival curves of patients admitted in 2019 and 2020 follow-up during 15 moths after admission. No significant differences in survival rates among the two groups was observed (Log-Rank *p* = 0.124).

The mortality of both groups was followed-up for 15 months after the admission day. The data obtained in patients admitted in 2020 showed a trend towards a reduced mortality compared to patients admitted in 2019 (32.3% vs. 38.0%, respectively, Supplementary Figure S1) although no statistical significance (*p* = 0.217) between study periods was reached. A trend towards higher mortality in higher tumor stages (*p* = 0.067) was observed as expected, with tumor staging not accounting for the slight decrease in mortality observed in the post-lockdown cohort. In the same line, the type of treatment did not influence mortality (*p* = 0.417) (Supplementary Table S3). The Log-Rank test showed no differences in survival rates among the two groups (Log-Rank *p* = 0.124; Figure 5 and Supplementary Table S4 include Cox regression test adjusted by variables). Patients with MUST score ≥ 2 showed higher mortality than patients without risk or malnutrition (*p* < 0.001, Supplementary Table S5).

## 4. Discussion

The GLIM [7] established a consensus for the identification and endorsement of criteria for the diagnosis of malnutrition in clinical settings including supportive proxy measures of inflammation, such as CRP, albumin, or pre-albumin. At the same time, the MUST was included as one of the best validated malnutrition screening tools, in line with the ESPEN criteria for the definition of malnutrition [25]. Inflammation contributes to malnutrition through associated anorexia and decreased food intake as well as altered metabolism with elevation of resting energy expenditure and increased muscle catabolism [26]. CRP, because of its sensitivity, specificity, and reproducibility in hospital laboratories, is most commonly used to assess the magnitude (whether acute or chronic) of the systemic inflammatory response. Indeed, the magnitude of the increase in CRP concentrations has been shown to be associated with poorer survival in patients with cancer, particularly in patients with advanced disease [27]. Contrary to the working hypothesis, the patients admitted to the hospital during the first 6 months after the lockdown presented a better nutritional status as evidenced by the MUST than patients admitted during the previous year, mainly due to significant differences in the lower percentage of weight loss. In addition, patients admitted during the post-lockdown period exhibited a statistically significant increase in circulating albumin concentrations and decrease in CRP levels, further supporting an improvement of their inflammatory state. These findings cannot be attributed to type of treatment since no differences between both periods were observed. The prevalence of patients with advanced stages (III and IV) in both groups justifies the high mortality found in both groups of the cohort. The significant difference in tumor stage distribution may be influencing the small tendency on mortality numbers between years in relation to the lower MUST score and lower inflammatory state observed in patients admitted in 2020. The fact that the hospital is a reference center for treatment of onco-hematological disease makes the prevalence of advanced cancer higher as compared to other hospitals. Despite other studies reflecting less use of nutrition support due to the fear of contagion during the pandemic [28], in the present study, no differences in the percentage of patients requiring nutrition support in both time periods were observed.

The extraordinary situation experienced during the lockdown increased the consumption of specific foods by the fear of a possible shortage. Spanish studies detected changes in dietary patterns throughout the 3 months that lasted during the confinement [29]. Food purchase in Spanish households changed abruptly since the beginning of the lockdown in March 2020, largely due to the increase in the intake of some foods such as bread, flour, and alcohol. The daily energy intake during the SARS-CoV-2 lockdown was 2509 kcal, which represented a 6% increase with respect to 2019, reflecting a 27% increase over the recommended value [30]. The combination of increased food intake and decreased physical activity was the main explanation for the weight gain observed during Spain’s COVID-19 lockdown response [31].

On the one hand, malnutrition may be a consequence of the oncological treatment-associated toxicity itself or surgery. On the other hand, the lack of an adequate nutritional state puts the patients at higher risk of developing toxicities, and it is one of the major causes of poor tolerance to therapies [32]. The national registry of the Spanish Society of Medical Oncology (SEOM) reflects that the number of patients treated in day hospitals decreased by 14% during the lockdown, the number of patients treated with chemotherapy decreased by 9.5%, and those treated with radiotherapy diminished by 5%. Oncological therapies, such as surgery, radiation therapy, and drug therapies, can play a role in the development of malnutrition and metabolic alterations in cancer patients [33]. While the interruption or delay of oncological treatments could contribute to explain the improvement of the nutritional state during lockdown [34], in the present study, the oncological treatments were not suspended.

The International Agency for Research on Cancer (IARC) established that overweight and obesity are associated with increased cancer incidence for more than 10 types of cancer [35,36]. However, published studies paradoxically observed the greater survival of oncological patients in association with overweight and obesity [37,38,39,40]. The analysis of body composition, the measurement of weight loss, and development of cancer cachexia may explain this ”obesity paradox” [41]. The measurement of body composition with differences between obesity with an excess of fat mass or obesity, sarcopenic obesity, and overweight with optimal level of fat-free mass is necessary to apply a more precise classification that explains the obesity paradox [42,43,44]. The secretion of a pleiad of adipokines and growth factors by the adipose tissue may further contribute to the obesity paradox [45,46,47]. In addition, the well-known cancer cachexia disorder is associated with advanced cancer, reflecting a poor nutritional status [48].

The lockdown has highlighted the need to develop nutritional interventions that reverse the negative effects of disease-related malnutrition in the hospital setting [49]. The lockdown improved the nutritional status of the patients admitted to the hospital, with a greater tendency to a higher BMI due in part to the confinement itself that forced staying at home, increasing food intake, and decreasing energy demands, which seems to protect them by reducing slightly the mortality. For older populations, being overweight was not found to be associated with an increased risk of mortality. However, there was an increased risk for those at the lower end of the recommended BMI range for adults [50].

Further studies analyzing the real food intake of oncological patients are needed because previous studies have suggested potential sources of natural products with an important role on inflammation in cancer and COVID-19 patients [51,52,53].

Some potential sources of bias of this study should be pointed out. First, it was not possible to obtain all the tumor stages of the patients because,, in some cases patients came from hospitals without electronic clinical record-sharing systems. Second, dietary patterns and physical activity during the lockdown that could influence nutritional status at admittance were not registered. Third, due to the pandemic situation, it was not possible to collect data on the real intakes of patients since the food remains were eliminated without further manipulation. Fourth, the high variability in combinations of chemotherapy drugs of the patients undergoing polytherapy (the majority of patients) makes it difficult to take into account the potential influence of each type of drug on the nutritional status. Finally, this study includes patients from a single private hospital, probably from a socioeconomic status that allowed access to a variety and amount of food that lead to eating more during the lockdown. On the other hand, strengths of the study are, first, the complete nutritional assessment carried out of the cancer patients in the first 24 h of admission despite the extraordinary situation, which allowed the classification of the nutritional status of oncological patients according to the GLIM criteria. Second, maintaining the oncological treatment during the lockdown allowed including a significant number of patients. In addition, the characteristics of a private hospital that continued its activity beyond the COVID-19 wards enabled the analysis of patients in a different clinical setting.

## 5. Conclusions

The SARS-CoV-2 lockdown improved the nutritional status of cancer patients at admission, with a decrease in the percentage of weight loss and CRP concentrations together with an increase in albumin levels compared to oncological patients admitted the previous year in a single center. The better nutritional status was associated with a slight but not significant reduction in mortality in patients admitted after the lockdown. Measurement of biochemical variables and malnutrition risk maybe helpful to anticipate changes in the nutritional status of patients with cancer. Future clinical trials of natural products with an important role on inflammation in cancer are necessary to improve the nutritional status of oncologic patients.

## Figures and Tables

**Table 1:** MUST five-step screening tool to identify adults who are malnourished or at risk of malnutrition.

Steps		
**Step 1: BMI Score** **(kg/m^2^**) >20: Score 0 18.5–20.0: Score 1 <18.5: Score 2	**Step 2: Weight loss score** Unplanned weight loss in past 3–6 months <5.0%: Score 0 5.0–10.0%: Score 1 >10.0%: Score 2	**Step 3: Acute disease effect score** If patient is acutely ill, and there has been or is likely to be no nutritional intake for > 5 days Score 2
**Step 4: Overall risk of malnutrition (add scores together to calculate overall risk of malnutrition):** Score 0: Low Risk.Score 1: Medium Risk. Score ≥ 2: High Risk.		
**Step 5: Management guidelines according risk** Low Risk, routine care—unless major clinical deterioration expected. Medium Risk, observe or treat if approaching high risk or if rapid clinical deterioration anticipated. High Risk, treat unless detrimental or no benefit from nutritional support expected e.g., imminent death.		

**Table 2:** Clinical characteristics of patients in the two study periods.

		2019	2020 Post-Lockdown	*p*
*n*		440	288	
Age (years)		62.3 ± 14.7	60.4 ± 16.5	0.103
Sex	Male	256	170	0.821
	Female	184	118	
LOS (days)		12.2 ± 12.4	13.1 ± 13.1	0.363
Weight (kg)		70.3 ± 14.7	72.1 ± 17.1	0.143
BMI (kg/m^2^)		24.8 ± 4.6	25.2 ± 4.7	0.321
Albumin (g/dL)		2.7 ± 0.6	3.0 ± 0.6	**<0.001**
Prealbumin (mg/dL)		15.0 ± 9.4	16.0 ± 7.7	0.604
Cholesterol (mg/dL)		136.5 ± 56.6	126.0 ± 52.8	0.447
Lymphocytes (10^9^ cells/L)		1.1 ± 0.8	1.2 ± 1.3	0.621
CRP (mg/dL)		7.17 ± 8.11	5.92 ± 7.27	**0.005**
MUST	BMI score	0.2	0.2	0.867
	Weight loss score	0.2	0.1	**0.032**
	Acute disease effect	0.2	0.1	0.262
	Overall risk	0.6	0.4	**0.042**

LOS, length of hospital stay; CRP, *C*-reactive protein. Data presented as mean ± SD. CRP concentrations were logarithmically transformed for statistical analysis due to non-normal distribution. Differences between periods were analyzed by two-tailed unpaired Student’s *t*-tests. Gender distribution was assessed by χ^2^ analysis. Bold *p*-values indicate statistically significant differences.

**Table 3:** Tumor stage and treatment of patients included in the study.

Stage					*p*
	I	II	III	IV	
2019	0.3%	3.8%	14.1%	81.8%	0.001
2020	6.2%	6.2%	17.1%	70.5%	
**Treatment**					
	**Monotherapy**	**Polytherapy**	**Radiotherapy**	**Surgery**	
2019	22.3%	74.1%	2.7%	0.9%	0.030
2020	14.6%	78.8%	4.2%	2.4%	

## Data Availability

The data presented in this study are available on reasonable request from the corresponding author.

## Supplementary Materials

The following supporting information can be downloaded at: https://www.mdpi.com/article/10.3390/nu14132754/s1. Table S1, Distribution of types of treatment depending on mechanism of action in both time periods; Table S2, Distribution of tumor staging according to MUST score and year; Table S3, Distribution of types of treatment depending on mechanism of action in relation with mortality; Table S4, Cox-regression analysis regarding the influence of year of admission, stage, treatment, sex, age, MUST score and BMI on mortality; Table S5, Distribution of mortality according to MUST score; Figure S1, Distribution of mortality rates of patients admitted in 2019 and 2020 post-lockdown.
