# Supplementary material for: Impact on the Nutritional Status and Inflammation of Patients with Cancer Hospitalized after the SARS-CoV-2 Lockdown
Source: Nutrients. 2022 Jul 2;14(13):2754. doi: 10.3390/nu14132754 (PMC9268830; doi:10.3390/nu14132754)
Supplement: Supplementary file 1 [file nutrients-14-02754-s001.zip › nutrients-1761786-supplementary.pdf]

Table S1. Distribution of types of treatment depending on mechanism of action in both time periods.

	Monoclonal antibodies	Alkylating agents	Platinum derivatives	Antimetabolite agents	Topoisomerase inhibitors	Microtubule inhibitors	Others	Polytheraphy	*p*
2019	8.3%	3.0%	0.7%	0.9%	0.9%	3.9%	7.8%	74.5%	0.158
2020	7.1%	0.7%	1.4%	1.1%	0.0%	2.1%	7.4%	80.2%	

Table S2. Distribution of tumor staging according to MUST score and year.

		Stage I	Stage II	Stage III	Stage IV	*p*
2019	MUST < 2	0.4%	3.9%	14.9%	80.7%	0.798
	MUST ≥ 2	0.0%	3.2%	11.1%	85.7%	
2020	MUST < 2	6.8%	7,8%	18.4%	67.0%	0.283
	MUST ≥ 2	3.3%	0.0%	11.5%	84.6%	
Total	MUST < 2	2.4%	5.1%	16.0%	76.4%	0.305
	MUST ≥ 2	1.1%	2.2%	11.2%	85.4%	

Table S3. Distribution of types of treatment depending on mechanism of action in relation with mortality.

	Monoclonal antibodies	Alkylating agents	Platinum derivatives	Antimetabolite agents	Topoisomerase inhibitors	Microtubule inhibitors	Others	Polytheraphy	*p*
Survived	6.1%	2.2%	0.7%	0.9%	0.7%	3.3%	8.3%	77.9%	0.417
Death	10.7%	1.9%	1.5%	1.1%	0.4%	3.1%	6.5%	74.8%	

Table S4. Cox-regression analysis regarding the influence of year of admission, stage, treatment, sex, age, MUST score and BMI on mortality.

	Hazard Ratio	Std. Err.	z	P < /z/	95% C.I.	
Year	1.279	0.213	1.48	0.140	0.923	1.773
Stage	1.401	0.174	2.71	0.007	1.098	1.788
Treatment	1.153	0.223	0.74	0.462	0.789	1.685
Sex	1.139	0.1772	0.84	0.401	0.840	1.545
Age	1.018	0.006	3.03	0.002	1.006	1.031
MUST score	1.049	0.0780	0.64	0.522	0.907	1.213
BMI	0.998	0.0189	-0.09	0.931	0.962	1.036

Table S5. Distribution of mortality according to MUST score.

	Survived	Mortality	*p*
MUST < 2	67.8%	32.2%	<0.001
MUST ≥ 2	45.7%	54.3%	

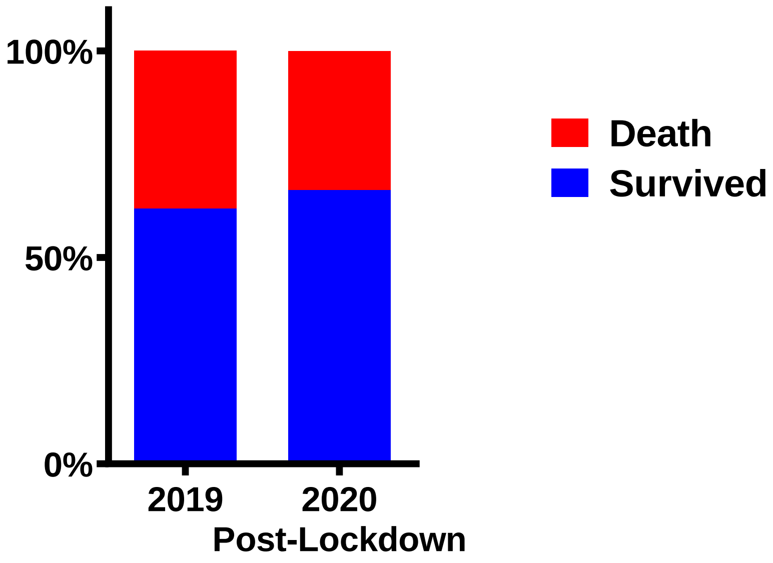

Figure S1. Distribution of mortality rates of patients admitted in 2019 and 2020 post-lockdown. Differences between time periods were computed by χ^2 analysis.
